# Knowledge, attitudes and behaviors of family physicians about childhood vaccinations that are not in the routine vaccination schedule: a cross-sectional study

**DOI:** 10.1017/S1463423622000688

**Published:** 2023-01-09

**Authors:** Duygu Avcı, Celal Kuş, Raziye Şule Gümüştakim, Duygu Ayhan Başer, Mustafa Emre Eryilmaz

**Affiliations:** 1 Gaziantep Oğuzeli State Hospital, Department of Family Medicine, Gaziantep, Turkey; 2 Kahramanmaraş Sütçü İmam University School of Medicine, Department of Family Medicine, Kahramanmaraş, Turkey; 3 Hacettepe University School of Medicine, Department of Family Medicine, Ankara, Turkey; 4 Kahramanmaraş Andırın State Hospital, Department of Family Medicine, Kahramanmaraş, Turkey

**Keywords:** family physicians, HPV, meningococcus, rotavirus, vaccine

## Abstract

**Aim:**

In our country, there are childhood vaccinations that are not included in the routine vaccination schedule and that families have to buy and have for a fee. In addition to income level, family physicians’ recommendations also play a major role in getting these vaccines.

Our study was planned to determine the level of knowledge, attitudes and behaviors of family physicians about rotavirus, HPV and meningococcal vaccines, which are not included in the routine vaccination scheme of the Ministry of Health.

**Materials and Methods:**

Our cross-sectional and descriptive study was carried out between May and July 2019. The population of our study consists of approximately 30 000 family physicians working as Family Physicians in Turkey. When the sample size is calculated with 5% margin of error and 95% confidence interval, it turns out to be 381. A 15-question questionnaire prepared by scanning the literature and including socio-demographic characteristics was presented to the participants. The Likert scale, which includes 12 questions about rotavirus, meningococcus, HPV and vaccines developed for these microorganisms, was administered to physicians either face-to-face or via the internet. In our study, the statistical significance level was accepted as *P* < 0.05, and the SPSS statistical package program was used in the calculations.

**Results:**

81 Research Assistants, 62 Family Medicine Specialists and 234 Family Physicians participated in our study, and the participants were determined by simple random sampling method. The mean age of the participating physicians was 37.96 ± 9.3 (min: 25 and max: 68). 50.9% of the physicians were women, 79.8% were married, 85.1% were in the city center, and 62.1% were practicing family medicine as general practitioners. 74.82% of the participating physicians recommend rotavirus and 56.2% HPV vaccines to their patients. 10.6% (40 people) of the physicians participating in our study did not recommend any of the rotavirus, HPV, meningococcal, influenza and adult pertussis vaccines to their patients. In the evaluation of the reason for this, 58.7% (27 people) of physicians who did not recommend special vaccines state that they did not recommend vaccines because they are not included in the routine vaccination schedule of the Ministry of Health. Another important reason was that the vaccines are paid (30.4%, 14 people). To the question of having sufficient information about special vaccines that are not included in the routine vaccination schedule, 26% of the participants stated that they have sufficient knowledge, and 56.5% stated that they have partial knowledge. The Likert knowledge questions total score of those who recommended at least one vaccine to their patients was significantly higher than those who did not recommend it at all. Likert knowledge questions total score of those who had at least one vaccination was significantly higher than those who never had it (*P* = 0.001).

**Conclusion:**

In general, as the level of knowledge about private vaccines decreases, the rates of self-vaccination, recommending it to their patients, and asking it to be included in the national vaccine schedule decrease. For this reason, increasing the knowledge of physicians about vaccines not included in the national vaccination schedule will contribute to the dissemination of vaccines, thus increasing immunity and reducing mortality and morbidity.

## Introduction

A vaccine is a biological compound that helps to provide immunity against a certain disease (Kara, 2017). The vaccine contains substances similar to a disease-causing microorganism and is usually obtained from attenuated or killed forms of the microorganism, toxins of the microorganism or related surface proteins. The vaccine stimulates the immune system to detect the substance applied to the body as foreign, to destroy it and to remember it when it is encountered again. In this way, the microbes will be familiar to the immune system when it encounters it again, so it will be easier to fight this microbe and, as a result, to be destroyed (Kara, 2017). Countries around the world have national vaccination programs that have been modified in line with the recommendations of organizations such as the World Health Organization (WHO) and are currently used. In Turkey, there is a routine immunization protocol under the control of the Ministry of Health. Currently, vaccines for hepatitis A and B, poliomyelitis, measles, rubella, mumps, tuberculosis, diphtheria, pertussis, tetanus, varicella, conjugated pneumococcus and haemophilus influenza type B are included in the national vaccination program of our country (Bakanlığı, 2021). Apart from these vaccines, there are various vaccines such as meningococcus, rotavirus, human papillomavirus (HPV), influenza virus, which are not included in the national vaccination program but are licensed for use.

Rotavirus is one of the leading causes of severe pediatric diarrhea in the world, causing ∼125 000–200 000 deaths in children under 5 years of age each year (Troeger et al., 2018; GBD Diarrhoeal Diseases Collaborators, 2017; Tate et al., 2016). Although the rotavirus burden has decreased considerably in the last decade, it still accounts for the majority of diarrheal deaths in children younger than 5 years of age and is associated with 130 000 deaths annually. Rotavirus was the third leading pathogen associated with death in 2016, after malaria and pneumonia. Approximately 40% of children younger than 5 years of age experienced rotavirus diarrhea in 2016, with large differences in incidence and mortality of this infection between high-income and low-income countries (Troeger et al., 2018).

HPV is one of the sexually transmitted diseases and is a virus that can cause anogenital and oropharyngeal infections in men and women. With high-risk HPV genotypes, permanent infections can develop and almost all cervical cancers develop in this way. HPV 16 and 18, which are high-risk HPV genotypes, are responsible for approximately 70% of cervical cancers worldwide. Of the other types, HPV types 31, 33, 45, 52 and 58 are held responsible for 20%. In addition, HPV types 16 and 18 are responsible for 90% of anal cancers and a significant portion of oropharyngeal, vulvar, vaginal and penile cancers. In addition, HPV types 6 and 11 are responsible for 90% of anogenital warts (Post, 2019). In the cancer statistics of Turkey published in 2014, the prevalence of cervical cancer in Turkey is given as 2%. The prevalence of HPV types 16 and 18 in Turkey has been reported to be 4.7%, and the prevalence of HPV in cervical cancer has been reported as 67.6% (Selçuk and Yanıkkerem, 2018). Vaccines have been developed to prevent the acquisition of HPV infection and to prevent HPV-related diseases that may develop afterward (Post, 2019).

Meningococcal diseases, especially meningococcal meningitis, are one of the most devastating diseases for a society or an individual (Post, 2019). Neisseria meningitidis generally affects healthy young individuals and is a microorganism that can cause death within hours (Post, 2019).

Our study was planned to determine the level of knowledge, attitudes and behaviors of family physicians about rotavirus, HPV, meningococcal vaccines other than the routine vaccination scheme of the Ministry of Health.

## Material and method

Our cross-sectional and descriptive study was carried out between May and July 2019. The universe of our study consists of approximately 30.000 family physicians working as Family Physicians in Turkey. When the sample size was calculated with 5% margin of error and 95% confidence interval, it turns out to be 381. 81 Research Assistants, 62 Family Medicine Specialists, 234 Family Physicians participated in our study, and the participants were determined by simple random sampling method.

Ethics committee approval was obtained for the study from the Clinical Research Ethics Committee of Kahramanmaraş Sütçü İmam University Faculty of Medicine, with the decision dated 05.05.2019 and numbered 14. A 21-question questionnaire prepared by scanning the literature, including socio-demographic characteristics, was applied to the participants after their consent. Our questionnaire consists of 6 questions in which socio-demographic characteristics are questioned, 13 items in which attitudes and behaviors are evaluated, and 19 statements and 2 information questions aiming to measure the level of knowledge about these vaccines. The Cronbach alpha value of our questions was calculated as 0.845, and it is quite strong. The Likert scale, which includes 19 questions about rotavirus, meningococcus, HPV and vaccines developed for these microorganisms, was administered to physicians either face-to-face or via the internet. In our study, socio-demographic characteristics and independent variables were determined and asked about age, gender, marital status, place of work and accordingly title, duration of work, having a child, vaccination status for their children, self-vaccination status, and the status of recommending vaccination to their patients. As dependent variables, the status of recommending the vaccine to their patients and their belief that the vaccine should be included in the national vaccination schedule were determined and asked to the participants.

## Statistical analysis

SPSS 22.0 analysis program was used to evaluate the data. Descriptive statistics are presented as mean (±) standard deviation, median (min-max), frequency distribution and percentage. Chi-square test or Fisher’s exact test was used to compare categorical variables. When a significant difference was detected in comparisons with at least one variable having more than 2 categories (comparisons other than 2 × 2), the groups were compared in pairs to determine the source of the difference, and Bonferroni correction was applied to identify the groups with difference. Conformity of continuous variables to normal distribution was examined using visual (histogram and probability charts) and analytical methods (if n ≥ 50; Kolmogorov-Smirnov test, if n < 50; Shapiro-Wilk test). For the variable found to fit the normal distribution, Student’s t-test was used for statistical significance between two independent groups. One-way ANOVA was used as a statistical method among three or more independent groups found to have a normal distribution. Tukey or Tamhane’s T2 test results were used according to the homogeneity of the variances of the groups in post hoc multiple comparisons to determine the source of the significant differences between three or more independent groups. Statistical significance level was accepted as *P* < 0.05.

## Results

The mean age of 377 family physicians participating in our study was 37.96 ± 9.3, and the median was 36 (min: 25 and max: 68). Of the physicians, 50.9% were women, 79.8% were married, 85.1% were in the city center, and 62.1% were practicing family medicine as general practitioners. The length of service of physicians in the profession was 12.54 ± 9.30 (min:1 and max:40).

Of the participants, 74.8% were recommended rotavirus, 67.9% were recommended Meningococcus, and 56.2% were recommended HPV. Most recommended another vaccine was the influenza vaccine with 59.4%. Most stated reasons for not recommending the vaccine were not being in the routine vaccination scheme of the Ministry of Health (58.7%), while the second most stated reason was paid for the vaccines (30.4%).

Of the participants, 274 had children, and 42.3% vaccinated their children for Rotavirus, 33.6% for Meningococcus, and 24.5% for Influenza. Only 8% of the physicians vaccinated their children against HPV, and 36.9% of them did not vaccinate their children with any special vaccines. Of the 103 physicians who do not have children stated that if they had a child, 84.5% of them would vaccinate for Rotavirus, 78.6% for Meningococcus, 66.0% for HPV, and 52.4% for influenza. Table 1 shows distribution of vaccines recommended by physicians.

**Table 1.** Physicians’ vaccination status

	n (%)
***Vaccination status (n = 377)***	
Vaccinated with at least one vaccine	207 (54.9)
Never vaccinated	170 (45.1)
***Own vaccination status (n = 377) #***	
Influenza	173 (45.9)
Meningococcus	42 (11.1)
HPV	31 (8.2)
Adult pertussis	11 (2.9)
None of them	170 (45.1)
***Thinking about getting vaccinated (n = 214)***	
Yes	100 (46.7)
No	114 (53.3)
***Distribution of vaccines they are planning to do(n = 100) #***	
HPV	62 (62.0)
Influenza	54 (54.0)
Meningococcus	43 (43.0)
Adult pertussis	14 (14.0)
***Reasons for not getting vaccinated for those*** ***who do not have any vaccinations (n = 118) #***	
I did not have it done because the ministry of health is not in the routine vaccination scheme.	52 (44.1)
I didn’t get it done because I missed the time (Influenza)	32 (27.1)
I didn’t do it because it’s paid.	24 (20.3)
I didn’t believe in its protection	15 (12.7)
I didn’t do it because of the side effects	14 (11.9)
I didn’t have enough information about vaccines	10 (8.5)

n: Number of patients; %: Column percentage; #There is more than one answer per participant, and the percentage is calculated on the number of patients.

While only 26.0% of the participants stated that they had sufficient knowledge about special vaccines, 56.5% stated that they had partial knowledge. The distribution of the participants’ knowledge about specific vaccines is shown in Table 2.

**Table 2.** Distribution of participants’ knowledge of specific vaccines

n (%)	
***The state of thinking that you have enough knowledge about specific vaccines (n = 377) #***	
Yes, I Have Enough Knowledge	98 (26.0)
I have partial knowledge	213 (56.5)
No, I Don’t Have Enough Knowledge	66 (17.5)
***Where to obtain information for those who have sufficient knowledge about specific vaccines (n = 247) #***	
Guidelines	139 (56.3)
In-service training	137 (55.5)
Congresses	81 (32.8)
Social media	48 (19.4)
Television	3 (1.2)
***Places where those who do not have enough information about special vaccines want to get information (n = 266) #***	
In-service training	236 (88.7)
Congresses	144 (54.1)
Social media	30 (11.3)
Television	11 (4.1)
I do not want to receive information	4 (1.5)
***The earliest and latest weeks of administration of the first dose of rotavirus vaccines. (n = 346)***	
The earliest is 4 weeks. the latest is 17 weeks, 6th day	44 (12.7)
Earliest 6 weeks, latest 14 weeks, 6th day (Correct answer)	118 (34.1)
Earliest 8 weeks, latest 12 weeks	125 (36.1)
Earliest 10 weeks 6 days latest 15 weeks 6 days	27 (7.8)
Earliest 16 weeks, latest 32 weeks	32 (9.2)
***Please tick the applications that you think are correct for the age ranges where the HPV vaccine is applied (n = 342) #***	
HPV vaccine is recommended for girls at the age of 11-12 (before the first sexual intercourse, if possible).	271 (79.2)
13–26 years old catch-up vaccine is applied in girls.	156 (45.6)
HPV vaccine is recommended for men 11-12 years old (before first sexual intercourse if possible)	135 (39.5)
Catch-up vaccine for men is administered between the ages of 11-21.	78 (22.8)
There is no upper limit for age for vaccination.	125 (36.5)
***Frequency of recommending meningococcal vaccine to non-risk age group (n = 370) #***	
6 weeks or more	65 (17.6)
0–2 years	90 (24.3)
0–5 years	70 (18.9)
11–18 years (Correct answer)	23 (6.2)
2–55 years	98 (26.5)
I do not recommend	95 (25.7)

n: Number of patients; %: Percent; #There is more than one answer per participant, and the percentage is calculated on the number of patients.

Of the family physicians, 69.7% thought that Rotavirus vaccine, 69.4% of them thought that Meningococcus vaccine, and 61.7% of them thought that HPV vaccine should be included in the national vaccination schedule. The distribution of the answers given by the participants to the suggestions asked about the vaccines not included in the routine vaccination schedule is presented in Table 3.

**Table 3.** Distribution of the responses of the participants to the suggestions asked about vaccines not in the routine vaccine schedule

	Agreed*	Not agreed*	No idea
(n = 377)	n (%)		
Rotavirus vaccine comes in two varieties, RotaTeq and Rotarix.	*309 (82.0)*	7 (1.9)	61 (16.2)
Rotavirus vaccines are administered orally	*317 (84.1)*	29 (7.7)	31 (8.2)
Rotavirus is the most important cause of severe gastroenteritis, which causes infant and child deaths.	*332 (88.1)*	26 (6.9)	19 (5.0)
Recommended scheme Rotarix 2-4th month, RotaTeq 2-4-6. month	*284 (75.3)*	24 (6.4)	69 (18.3)
Rotavirus can be prevented with water hygiene and sanitation	199 (52.8)	*146 (38.7)*	32 (8.5)
The last dose of both vaccines for rotavirus can be administered at the latest in the 34th week and 6 days.	*107 (28.4)*	121 (32.1)	149 (39.5)
There are two types of meningococcal vaccines, conjugated and polysaccharide.	*275 (72.9)*	37 (9.8)	65 (17.2)
There are A, C, W, Y serotypes in the meningococcal vaccines used in Turkey.	*187 (49.6)*	35 (9.3)	155 (41.1)
Current meningococcal vaccines provide lifetime protection	109 (28.9)	*150 (39.8)*	118 (31.3)
Meningococcal W-type is associated with pilgrimage and Umrah	*195 (51.7)*	17 (4.5)	165 (43.8)
The most common meningococcal serotypes in our country are W and B serotypes.	*171 (45.4)*	29 (7.7)	177 (46.9)
MENVEO, MENECTRA, NIMENRIX vaccines containing A, C, W, Y serotypes are currently in use in our country.	*195 (51.7)*	23 (6.1)	159 (42.2)
BEXSERO containing meningococcal B serotype is still not in use in our country.	63 (16.7)	*126 (33.4)*	188 (49.9)
HPV 16 and 18 are not associated with cancer	35 (9.3)	*291 (77.2)*	51 (13.5)
There are bivalent (CERVARIX) and quadrivalent (GARDASIL) vaccines against HPV virus.	*233 (61.8)*	11 (2.9)	133 (35.3)
Gardasil at 0-2-6 months CERVARIX is administered at 0-1-6 months	*133 (35.3)*	27 (7.2)	217 (57.6)
HPV quadrivalent vaccine targets HPV 6, 11, 16, 18, HPV bivalent vaccine targets HPV 16 AND 18	*230 (61.0)*	16 (4.2)	131 (34.7)
Regular cervical scans are not necessary for vaccinated women	25 (6.6)	*304 (80.6)*	48 (12.7)
Breastfeeding is not contraindicated by HPV vaccination.	*204 (54.1)*	35 (9.3)	138 (36.6)

n: Number of patients; %: Percent *: Correct answers are in italics.

There was no significant difference between considering the necessity of inclusion of the rotavirus vaccine in the national vaccination schedule according to gender, age, marital status, place of work and working status (*P* > 0.05), and a significant difference was found only between the length of service in the profession (*P* = 0.026). Considering that rotavirus should be included in the national vaccination schedule was significantly higher in those working for 10–19 years compared to those working for 30 or more years (*P* = 0.009).

No significant difference was found between socio-demographic parameters and considering the need for HPV vaccine and adult whooping cough vaccine to enter the national vaccination schedule (*P* > 0.05).

In terms of meningococcal vaccine, a difference was found with gender, working year in the profession and working status, and it was observed that women, research assistants and those working in the profession between 10 and 19 years were significantly more likely to want the meningococcal vaccine to be included in the national vaccination schedule (respectively *P* < 0.001; *P* = 0.045; *P* < 0.001).

Single people stated that they wanted the influenza vaccine to be included in the vaccination schedule significantly more than married people and family physicians compared to family medicine research assistants (*P* = 0.024 and *P* = 0.009, respectively).

Women (*P* < 0.001), in those aged 25–29 years (*P* = 0.002), in those who work 10–19 years in the profession compared to those who work for 30 years or more (*P* = 0.005) were significantly more likely to want the Rotavirus vaccine to be included in the national vaccination schedule.

It was determined that HPV vaccine was recommended mostly by women, singles and family medicine research assistants, and this situation was found to be statistically significant (*P* = 0.007; *P* = 0.031; *P* < 0.001, respectively).

It was determined that Meningococcal vaccine was recommended mostly by women, 25–29 age group and family medicine research assistants, those who work in the profession for 10–19 years and this situation was found to be statistically significant (*P* < 0.001; *P* < 0.001; *P* = 0.002; *P* < 0.001; respectively).

It was determined that family medicine specialists recommended influenza vaccine significantly more than women and family physicians (*P* < 0.001 and *P* = 0.001, respectively). Adult pertussis vaccine was also recommended significantly more by family medicine research assistants (*P* = 0.007).

It was found that people who recommended HPV vaccine and influenza vaccine had these vaccines significantly more than those who did not (*P* = 0.004 and *P* < 0.001), this situation was not valid for meningococcal and adult pertussis vaccines (*P* > 0.05). Those who recommend rotavirus, HPV, meningococcal and influenza vaccines to their patients have their children vaccinated significantly and at a higher frequency (*P* < 0.001). Again, it was determined that female physicians vaccinated their children with special vaccines significantly more (*P* = 0.004).

Women compared to men (*P* = 0.001), 30–39 age group compared to 40–49 age group (*P* = 0.016), and those who work 10–19 years in the profession compared to those who work for 30 years or more (*P* = 0.005) stated that at least one vaccine should be included in the national schedule. Men, those aged 50 and over, and family medicine research assistants stated that they did not have enough knowledge about special vaccines compared to others, and this relationship was also found to be statistically significant (*P* = 0.013; *P* = 0.043; *P* = 0.001, respectively).

The comparison of the individual and total scores of the participants from the information questions is given in Table 4 and Table 5. It was observed that the HPV knowledge score was significantly lower in those aged 50 and over, those who did not recommend any vaccination, and those who did not have any vaccinations (Table 4). The same results were found in terms of total points (Table 5).

**Table 4.** Comparison of participants’ Rotavirus HPV and Meningococcal information questions according to correct knowledge

	Rotavirus Knowledge score	HPV Knowledge score	Meningococcus Knowledge score
	Mean ± SD	Mean ± SD	Mean ± SD
**Age**			
25–29	0.37 ± 0.49 (n = 91)	2.52 ± 1.33 (n = 90)	0.10 ± 0.30 (n = 92)
30–39	0.36 ± 0.48 (n = 119)	2.29 ± 1.34 (n = 119)	0.05 ± 0.22 (n = 121)
40–49	0.26 ± 0.44 (n = 102)	2.03 ± 1.27 (n = 99)	0.04 ± 0.21 (n = 112)
50 ve over	0.41 ± 0.50 (n = 34)	1.88 ± 1.20 (n = 34)	0.07 ± 0.25 (n = 45)
**p** ^**1**^	0.258	**0.024** ^**c**^	0.403
**Recommending Vaccines Not in the Vaccination Schedule to Children, Adolescents and Adults**			
Recommend at least one vaccine	0.35 ± 0.48 (n = 318)	2.28 ± 1.33 (n = 314)	0.07 ± 0.25 (n = 333)
Never recommend	0.21 ± 0.42 (n = 28)	1.71 ± 1.05 (n = 28)	0.00 ± 0.00 (n = 37)
**p** ^**2**^	0.108	**0.028**	**<0.001**
**Own vaccination status**			
Vaccinated with at least one vaccine	0.37 ± 0.48 (n = 192)	2.38 ± 1.37 (n = 190)	0.07 ± 0.25 (n = 203)
Never vaccinated	0.31 ± 0.46 (n = 154)	2.05 ± 1.23 (n = 152)	0.05 ± 0.23 (n = 167)
**p** ^**2**^	0.207	**0.019**	0.551

n: Number of patients; %: Percent; 1: One-Way Analysis of Variance; 2: t-test in Independent Groups; Mean: Mean; SD: Standard Deviation; c: In post hoc pairwise comparison, the difference is between 40–49 and 25–29 groups (*P* = 0.049).

**Table 5.** Comparison of the scores obtained from correct answers to Likert information questions according to some properties

	Likert Knowledge Questions Total Score	
	Ort±SS	p
**Age**		
25–29 (n = 92)	11.53 ± 3.07	0.001*^c^
30–39 (n = 123)	11.84 ± 3.92	
40–49 (n = 114)	10.75 ± 4.20	
50 and over (n = 48)	9.31 ± 5.06	
**Recommending Vaccines Not in the Vaccination Schedule to Children, Adolescents and Adults**		
Recommend at least one vaccine (n = 338)	11.55 ± 3.83	0.001**
Never recommend (n = 39)	7.28 ± 3.97	
**Own vaccination status**		
Vaccinated with at least one vaccine	12.04 ± 3.98	0.001**
Never vaccinated	9.98 ± 3.85	

n: Number of patients; %: Percent;*: one-way analysis of variance **: t-test in independent groups; In c:post hoc paired comparison, a difference was found between the 30–39 and over 50 age groups (*P* = 0.016).

## Discussion

One out of every ten family physicians participating in our study did not recommend any of the special vaccines. While the biggest reason why physicians did not recommend special vaccines in our study was that these vaccines were not included in the vaccine schedule, the other important reason was that these vaccines were paid. Physicians who did not vaccinate themselves and who did not plan to vaccinate themselves had the same reasons for not recommending the vaccine. Although this situation is compatible with the literature (Parashar et al., 2013), this problem can be solved by the Ministry by including these vaccines in the national vaccination schedule and by ensuring that the costs are covered by the state.

In most studies, vaccines were discussed one by one, and their knowledge levels were also evaluated separately. In a systematic review in which 60 studies were evaluated, it was found that correct answers given to items evaluating HPV knowledge varied between 22% and 95%, and correct answers given to items assessing HPV vaccine knowledge ranged between 17% and 91%. The level of knowledge of clinicians about HPV in men is lower than in women (Rosen et al., 2018). In a study conducted with gynecologists, pediatricians, family physicians and infection specialists in Lebanon, the HPV knowledge level was found to be 73% (Abi Jaoude *et al*., 2019). In a large-scale study conducted with healthcare professionals, HPV vaccine knowledge score was determined as 69.2% and attitude score was 5 on average (Trucchi et al., 2020). In a study conducted with gynecologists in Serbia, the knowledge of gynecologists was calculated as average. 98.3% of gynecologists thought that they needed additional training on this subject (Stamenkovic et al., 2017). In a study conducted with child health workers (doctors and nurses) in Sweden with an interval of 2 years, it was seen that the level of rotavirus knowledge was higher in the study performed 2 years later (Stenmarker et al., 2021).

About the special vaccines that are not included in the routine vaccination schedule, 26% of the participants state that they have sufficient information, and 56.5% have partial knowledge. Despite this, the frequency of giving correct answers to the questions regarding the time of recommendation for vaccines remained low in general. It is thought that the reason for this may be the variability in the frequency of in-service training for physicians. The vast majority (88.7%) of those who do not have sufficient knowledge stated that they want to overcome these deficiencies with in-service training. Based on our results, it can be said that the lack of knowledge about vaccines in our country is much higher than the studies in the literature. For this, it is necessary to support and update the training of family physicians, who are the first contact point in health, both in the assistantship process and in the process they work in the field.

According to the descriptive characteristics of the participants in our study, statistically significant results were obtained when the request for at least one vaccine to be included in the national vaccination schedule was compared, and female physicians want the addition of specific vaccines to the National Vaccine Program at a higher frequency than men. Similarly, in the results of our study, it was determined that female physicians recommended more vaccines. When compared by age, physicians aged 30–39 were more willing to add special vaccines to vaccination program than physicians aged 40–49. Similarly, the frequency of requesting the addition of at least one vaccine to the national vaccination program was found to be statistically significantly higher among physicians with 10–19 years of service, according to their professional education period, compared to those with 30 or more years of service. From this point of view, it can be concluded that people with a service period of more than 30 years think that the vaccination schedule is sufficient and that adding any of the special vaccines to the schedule is unnecessary. However, although many communities who have a say in this subject recommend adding the rotavirus vaccine to the calendar, it can be said that awareness should be raised with in-service training for physicians and these differences between groups should be evaluated.

In our study, it was concluded that female physicians both have more sufficient information and vaccinated their children more than male physicians, and this is because women do more research for their children on this subject, and therefore have more information, and as a result of all these, they can protect their children more based on their knowledge.

The total scores of knowledge questions of those who recommend at least one vaccine to their patients and those who have had at least one vaccine were significantly higher than those who did not recommend it at all and those who have never had it. From this point of view, it can be concluded that those who have correct knowledge about vaccines do not mind getting the vaccine themselves and therefore recommend it to their patients.

## Rotavirus vaccine

Prior to administration of rotavirus vaccines, more than 65% of children had at least one rotavirus diarrhea by age 5, and >40% of all-cause diarrhea hospitalizations worldwide were rotavirus-related (Burnett et al., 2018). Vaccination of infants against rotavirus is recommended globally in line with the recommendations of the Center for Disease Control and Prevention, WHO, American Academy of Pediatrics, American Academy of Family Physicians, European Pediatric Infectious Diseases Society and European Pediatric Gastroenterology Hepatology and Nutrition Society (Post, 2019). In 2009, it was recommended by the WHO that all countries include a live oral rotavirus vaccine in their routine infant immunization programs, and as of 2018, 101 countries included rotavirus vaccines in their national immunization programs. In countries where the rotavirus vaccine was administered, a 40% decrease in hospitalization rates due to rotavirus among children under 5 years of age and a 25% decrease in annual rotavirus diarrhea deaths worldwide were observed. The efficacy of oral rotavirus vaccines and their success in reducing mortality and hospitalization have been proven by randomized controlled studies and meta-analyses (Burnett et al., 2020; Clark et al., 2019). Although rotavirus vaccines are already in use in Turkey, they have not been included in the national vaccination program yet. It is important that family physicians, who are involved in the administration and follow-up of the vaccines included in the national vaccination program, have information about the special vaccines that are not included in the calendar.

Of the 377 family physicians included in our study, 74.8% recommend rotavirus vaccination, 42.3% report that they have their own child vaccinated against rotavirus, and 84.5% of the participants who do not have children would vaccinate if they had children. 69.7% of the participants thought that the rotavirus vaccine should be included in the national vaccination schedule. In a study conducted by Sperou et al. (2017) in 2017, 573 physicians supported future vaccines for pathogens that cause gastroenteritis, and in another study by O’Leary et al. (2013), 65% of family physicians in the USA were pediatricians. It was reported that 95% of them routinely recommend rotavirus vaccine. In a study conducted by Parlakay et al. (2020), it was stated that 82.8% of the physicians working in the third-level pediatric hospital recommended the vaccine. In another study conducted with 300 family physicians and 230 pediatricians, it was determined that the rotavirus vaccine was the most recommended vaccine (60.5%) by the physicians and requested to be included in the vaccine schedule (48.5%). It was observed that pediatricians recommended rotavirus, meningococcal and Tdap vaccines more than family physicians (Çataklı et al., 2018). In a study conducted with primary care physicians, it was found that approximately one-fifth of physicians recommended rotavirus vaccines to their patients, a very small portion (7.8%) vaccinated their own children, and only 39.4% thought that the vaccine should be added to the national schedule (Almış and Bucak, 2017). In four studies in a systematic review evaluating physicians’ perceptions of the rotavirus vaccine, it was observed that the vaccine was recommended more by pediatricians (70%–88%) than family physicians (46.1%–55%). Pediatricians perceived rotavirus vaccine as a need at a higher rate (66%–83%) compared to family physicians (28%) (Apte et al., 2018). In a study conducted in Belgium, it was concluded that the risk of incomplete vaccination with the rotavirus vaccine is higher when the vaccine is prescribed by family physicians compared to pediatricians (Braeckman et al., 2014). In a study conducted with Italian physicians, the rate of recommending rotavirus vaccines by physicians was found to be 57.4%, and physicians stated that they would recommend the vaccine to their patients at a much higher rate (81.1%) if the vaccine was free of charge (Valentin et al., 2017). Similarly, in our study, it was concluded that there was an increase in the frequencies of recommending the vaccine, and the frequency of physicians recommending the rotavirus vaccine increased, which is a pleasing development.

The frequencies of recommending the rotavirus vaccine in the physicians participating in our study were found to be higher than the frequencies of considering it to be included in the routine vaccination schedule. The differences in these frequencies guide us in the need for physicians to be further enlightened in terms of vaccine protection, immunity, cost-effectiveness and the contribution of these factors to health and economy.

In a study conducted with the data of 49 countries, it was reported that the rotavirus vaccine was recommended in 27 countries, this vaccine was included in the national immunity program in most countries in 2012 and 2014, and the vaccination costs were mostly covered by the government and insurance, including the families. It has been stated that the high cost of the vaccine is the biggest obstacle to the administration of the rotavirus vaccine. It has been observed that high vaccination costs and family reimbursement are significantly associated with a lower immunization rate. In addition, limited perception of the severity of this disease and timing of implementation by families, public health officials and doctors are listed as other barriers (Parashar et al., 2013). The results of our study were also found to be compatible with the literature, and the burden of vaccination costs on families also greatly affects physician recommendations. It may be a solution if an important vaccine such as rotavirus, which is included in the vaccination schedules of more than 100 countries in the world, is included in our national vaccination schedule and the costs are covered by the government.

It was observed that the participants did not have enough information about the route of transmission of rotavirus (correct response frequency 38.7%) and the last dose of the vaccine (correct response frequency 28.4%) in the questions about the propositions in which general information about vaccines was evaluated. The frequency of giving the correct answer to the question about the earliest and latest time to administer the first dose of the rotavirus vaccine was 34.1%. In this respect, it can be thought that physicians should be provided with the necessary methods and trainings to have a better command of the subject.

According to the descriptive features, the frequency of requesting the rotavirus vaccine in the national vaccination schedule of those with a service period of 10–19 years in the profession is statistically significantly higher than those with a service period of 30 years or more, but from this point of view, those who have been practicing medicine for a long time need in-service training in order to update their knowledge. It can be said that taking it to the forefront will have an important place in the vaccine recommendation part.

Although there was no significant difference according to the rotavirus knowledge score and the status of recommending at least one vaccine not included in the vaccination schedule, it was observed that there were deficiencies in the use of rotavirus knowledge in terms of recommending a vaccine. It was observed that those who gave wrong answers to the questions about the rotavirus vaccine thought that they did not have enough information anyway. The frequency of incorrect answers was found to be the highest among those who had partial knowledge (61.8%). Accordingly, it can be concluded that re-informing physicians about the subject will be an important step in correcting known misconceptions.

## HPV vaccine

HPV is one cause of the sexually transmitted diseases and is a virus that can cause anogenital and oropharyngeal infections in men and women (Post, 2019). While permanent infections can be seen with high-risk HPV genotypes, almost all cervical cancers develop in this way (Post, 2019). Vaccines have been developed to prevent the acquisition of HPV infection and to prevent HPV-related diseases that may develop afterward (Post, 2019). HPV vaccines are effective in preventing many HPV-induced cervical, anogenital, oral and respiratory diseases, including diseases such as cervical intraepithelial neoplasia and adenocarcinoma in situ (Post, 2019).

In the study of Allison et al., 60% of pediatricians and 59% of family physicians recommended HPV vaccine to girls aged 11-12. Recommendations for boys are much less (52% vs 41%). 84% of pediatricians and 75% of family physicians discuss HPV vaccination with the family at the 11- to 12- year-old visits. It was found that the physicians who had less discussion with the family on this issue were mostly family physicians, were male, were of the opinion that the HPV vaccine would not be accepted together with other vaccines, thought that the family would delay it and thought that the immunity would decrease (Allison et al., 2016).

In a study by Petrusek et al., it was observed that more than 95% of family physicians recommended HPV vaccine to men and women. It has been observed that vaccination recommendations have decreased in family physicians who provide service to the group exceeding the recommended age range (Petrusek et al., 2020). In a study involving pediatricians in 2011 (Özsurekçi, 2013), the frequency of participants who thought that the HPV vaccine should be included in the national vaccination schedule was found to be 70%. The reason for not considering was the cost of the vaccine in the foreground. In the study of Revanlı et al. (2016), in parallel with the study of Tolunay et al. (2014), it was determined that 59.5% of the physicians participating in the study recommended HPV vaccine to their patients. In the study of Tolunay et al. (2014), it was determined that the physicians who did not recommend the vaccine indicated the cost as the primary reason. In addition, in the study conducted by Sakanishi et al. (2018) in 2012 in Japan, it was determined that primary care physicians recommended the HPV vaccine at a frequency of 58.1%. In a study conducted with tertiary healthcare professionals, 80.2% of the participants were aware of the existence of the HPV vaccine, but only 30.5% stated that they or a relative had the HPV vaccine. 65.8% of the participants reported that they would agree to get HPV vaccine for themselves, 71.6% for their daughters and 59.5% for their sons. While 75.3% of the participants agree to be vaccinated if the cost of the vaccine is covered by the state, this rate drops to 54.3% if the cost of the vaccine is covered by them (Öz et al., 2019).

In a study conducted with 247 family physicians, when asked openly whether they recommend the HPV vaccine to their patients, 87 (35.2%) reported that they recommend the HPV vaccine to their patients. When the participants who did not recommend HPV vaccine were questioned why they did not recommend the vaccine, 101 (40.9%) of them did not have sufficient knowledge and experience about the vaccine, 61 (24%) found the vaccine expensive, 41 (16.6%) were not in the national vaccine schedule, 22 (8.9%) were not widely accepted in the society, 11 (4.5%) were not accepted by their colleagues, 6 (2.4%) because of side effects, 4 (1.6%) stated that it would increase risky sexual behaviors and cause social stigma. reported that they did not recommend vaccination because they thought (Aydın, 2019). When the opinions of the family physicians participating in the study about the addition of the HPV vaccine to the national vaccination schedule are evaluated; While 126 (51%) family physicians stated that they supported the addition of the vaccine to the national vaccination program, 18 (7.3%) stated that they did not, while 103 (41.7%) stated that they were undecided (Aydın, 2019).

In a study conducted with gynecologists, pediatricians, family physicians and infection specialists in Lebanon, although the cost of HPV vaccine is a common problem for both doctors (58.9%) and families (80.7%), less than half of the physicians see the fact that the HPV vaccine is not compulsory as a barrier in itself (45.9%). Approximately one-fourth of the physicians tend to always prescribe HPV vaccine to women, regardless of the pap smear results, and only a few (6%) of the physicians always recommend HPV vaccine to their male patients (Abi Jaoude *et al*., 2019).

In a systematic review in which 60 studies were evaluated, the average of intent ratios is 66.9 and the median is 73. In 2 studies examining intention by patient age, 13- to 18-year-olds (92%–96%) found higher intention than 11- 12-year-olds (73%–78%). In a study investigating clinicians’ intentions to recommend HPV vaccine by patient gender, 67% of clinicians aimed to recommend HPV vaccine only to female patients, and 14% aimed to recommend the vaccine equally to boys and girls. It was determined that higher knowledge levels, professional factors and beliefs and attitudes about HPV vaccines affected the clinician’s recommendation for vaccines. In many studies, it has been reported that the HPV vaccine is recommended less strongly than other vaccines by clinicians, that the HPV vaccine is recommended as ‘on-demand’, and that the absence of the HPV vaccine’s school entry requirement is believed to be an obstacle to making strong recommendations (Rosen et al., 2018).

In another study conducted with physician mothers, it was concluded that the branch of the mother affected the idea of having her child vaccinated and that mothers in family medicine, obstetrics and pediatrics wanted to have their children vaccinated significantly more. In the same study, in parallel with our study, it was observed that physicians who did not plan to have their children vaccinated against HPV did not recommend the vaccine to their patients at a higher rate. This situation was also found to be statistically significant. Again, it was determined that those who had family medicine, obstetrics and pediatrics branches significantly more recommended the HPV vaccine to their patients. Approximately two-thirds of physician mothers want the HPV vaccine to be added to the vaccination schedule (Döner Güner and Gözükara, 2019). In a study conducted with primary healthcare workers, 83% of family physicians recommend HPV vaccine to their patients, while 65% state that they would recommend HPV vaccine to their daughters (Özbakir et al., 2019). In another study conducted with 300 family physicians and 230 pediatricians, physicians recommend the HPV vaccine is 45.6%. Although the HPV vaccine is the least given to children by physicians, the most frequently administered vaccines are rotavirus and meningococcal vaccines. This is in line with the recommendations of the physicians (Çataklı et al., 2018).

In our study, this frequency was found to be 61.7%, which is higher than the study in Japan. In the study of Özsürekçi et al., 75% of the participants stated that they have or will have their own children vaccinated (Özsurekçi, 2013), while 56.2% of the 377 family physicians in our study recommend HPV vaccination, 8% said that they have their own children vaccinated with HPV. 66% of them reported that they would get the vaccine if they had a child. 8.2% of the participants in our study had HPV vaccination, and 62% of those who are considering vaccination after that state that they are considering HPV vaccination. While 11.8% of those recommending the HPV vaccine had it vaccinated, 3.6% of those who did not recommend it had it, and those recommending the HPV vaccine were found to have the vaccine more frequently. In addition, the frequency of HPV vaccination to their own children was found to be significantly higher in those who recommended the vaccine. In addition, Family Medicine Research Assistants recommended HPV vaccination more frequently than other physicians. The approaches regarding the recommendation of HPV vaccine in our study are approximately parallel to the frequencies in the literature. It is obvious that there is a need for further studies to increase these frequencies for the prevention of cervical cancer and other HPV-related diseases in the future.

In a large-scale study conducted with healthcare professionals, the majority of whom were Italian doctors, it was observed that 87% of them recommended the HPV vaccine. HPV vaccine knowledge score was determined as 69.2% and attitude score was 5 on average. Regarding the vaccination recommendations, almost all of them stated that they would vaccinate their pre-pubertal son/daughter against HPV and 87.1% of them were in favor of making the vaccine compulsory for them. The vaccine selection process is greatly influenced by parents’ attitudes and the cost of vaccines. It has been observed that attitudes differ significantly between health professionals and physicians, physicians have more positive attitudes than other health professionals (for example, nurses, health assistants and obstetricians), and they accept all suggested sentences more than other health professionals. It was determined that those with a good knowledge score gave more advice to adolescents, and there were significant differences in knowledge and attitudes among physicians, general practitioners/pediatricians and other health professionals (ie, nurses, health assistants, obstetricians) (Valentin et al., 2017).

In a study conducted with gynecologists in Serbia, the knowledge of gynecologists was calculated as average, and 98.3% of gynecologists think that they need additional training on this subject. The most frequently cited barriers to HPV vaccination are financial concerns (59.8%), and more than two-thirds of gynecologists are willing to recommend the vaccine (68.4%). Factors associated with gynecologists’ intention to recommend the vaccine include positive attitudes towards the vaccination of boys, negative attitudes towards frequent changes in recommendations and beliefs that vaccination will reduce condom use. Average knowledge level is 10.56 out of 15, and there is a statistically significant difference between knowledge levels and vaccine recommendations (Stamenkovic et al., 2017).

It has been observed that HPV knowledge score is inversely proportional with increasing age, not recommending the vaccine and not having it, and the knowledge score decreases in such participants. Considering the frequencies of correct answers given to the questions to determine the level of knowledge about HPV, it can be concluded that training activities aimed at providing information about the vaccine and HPV-related diseases will be beneficial in terms of vaccination and recommending the vaccine, and will increase the frequencies of vaccination even more.

Women recommended HPV vaccine at a higher frequency, and this may be due to the fact that women are more likely to be exposed to serious diseases such as cervical cancer, which can develop due to HPV, compared to serious HPV-related diseases in men, and this may affect women’s tendency to vaccination. In addition, single people suggested the vaccine significantly more, and it can be thought that this result may be caused by the uncertainty of the future spouse, HPV infection or immune status.

## Meningococcal vaccine

Meningococcal diseases, especially meningococcal meningitis, are one of the most devastating diseases for a society or an individual (Post, 2019). Neisseria meningitidis generally affects healthy young individuals and is a microorganism that can cause death within hours (Post, 2019). While many organizations such as WHO, ACIP and AAP recommend routine meningococcal vaccines (Post, 2019), meningococcal vaccines can also be used in epidemic control (Post, 2019).

Of the 377 family physicians, 67.9% recommended meningococcal vaccination in our study, 33.6% of them reported that they have their own child vaccinated against meningococcus, and 78.6% of the participants who do not have children would have the vaccine if they had children. 11.1% of the participants in our study stated that they had meningococcal vaccine, and 43% stated that they were considering getting the meningococcal vaccine. In a study conducted by Özdemir et al. [37], it was reported that 40.7% of the participating physicians recommended meningococcal vaccine to all their patients, 56.8% to their patients in the risk group, and 2.5% did not recommend the vaccine at all. The most important reason reported by those who did not recommend the vaccine is the cost of the vaccine [37]. In the same study, the frequency of those who had their own child vaccinated or were considering having it was found to be 80% [37]. Those who stated that the meningococcal vaccine should be included in the national vaccination schedule were 81.8% [37], which was much higher than the frequency in our study (69.4%). In conclusion, it was reported in this study that when physicians and families are well informed and the cost of vaccination is reduced, the tendency to have and recommend vaccination increases [37]. The same is valid for our study.

In a study conducted with 300 family physicians and 230 pediatricians, it was seen that pediatricians recommended rotavirus, meningococcal and Tdap vaccines more than family physicians. The rate of recommendation of meningococcal vaccine by physicians is 52.6%. The vaccines most frequently given by physicians to their children are rotavirus and meningococcal vaccines. This is in line with the recommendations of the physicians. It was observed that female physicians recommended Tdap vaccine more and younger physicians recommended pertussis vaccine more. The most important factor affecting the vaccination recommendations of the physicians was found to be the expensiveness of the vaccines (Çataklı et al., 2018).

In our study, the frequency of correct answers to the information questions about the meningococcal vaccine of the participants was determined as 6.2%, and it was concluded that the majority of them did not have sufficient knowledge about the vaccine. It was observed that the current meningococcal vaccine types were known by 72.9% of the participants, but the frequency of correct answers to the questions in which the vaccine types were detailed was around 50% or less. Those who recommended at least one vaccine had a significantly higher meningococcal knowledge score than those who did not. According to these data, there is a serious lack of information about meningococcal vaccine, and it can be concluded that the information to be given on this subject will increase the frequency of recommending the vaccine and therefore the vaccination frequencies.

In our study, it was seen that female physicians thought that meningococcal vaccine should be included in the National Vaccination Schedule more than men, and similarly, female participants recommended the vaccine to their patients more. Those who recommended the meningococcal vaccine to their patients were more likely to have their own child vaccinated. Looking at other descriptive information, it was determined that Family Medicine Research Assistants (77.8%) and those with 10–19 years of service in the profession (77.2%) thought that the meningococcal vaccine should be included in the National Vaccination Schedule. In addition, while Family Medicine Research Assistants recommend the vaccine to their patients the most, those who work in the profession for 30 years or more and the participants whose age is 50 and over are the least recommended. In the light of these data, it can be concluded that the use of more up-to-date information and training activities for vaccines by doctors who have worked in the profession of medicine for many years and who are over 50 years old will be an important step in increasing the frequencies of recommending the vaccine. It can be thought that these data will help in determining the physician groups that should be given priority in in-service training.

## Conclusion

As a result of our study, among the vaccines that are not included in the national vaccination schedule, the most recommended vaccine to their patients by the physicians participating in the study was determined as the rotavirus vaccine. Other recommended vaccines are, in order of frequency, meningococcal, influenza, HPV and adult pertussis vaccine. Approximately one-tenth of physicians did not recommend any of the vaccines that are not included in the national vaccination schedule to their patients. Among the reasons put forward by physicians who did not recommend vaccines, the most dominant one was that the vaccine is not included in the vaccine schedule of the Ministry of Health. The other most important reason was the cost of the vaccine. This is followed by hesitation due to possible side effects of the vaccine, not having enough information about vaccines and not believing in the protection of the vaccine. In general, as the level of knowledge about special vaccines decreases, the frequency of self-vaccination, recommending it to patients and asking it to be included in the national vaccine schedule, also decreases. For this reason, increasing the knowledge of physicians about vaccines not included in the national vaccination schedule will contribute to the dissemination of vaccines, thus increasing immunity and reducing mortality and morbidity.
